# Development of a novel rat model for pancreaticoduodenectomy

**DOI:** 10.1038/s41598-025-24072-x

**Published:** 2025-11-18

**Authors:** Yifei Yang, Zhiang Wang, Neng Tang, Liang Mao, Yudong Qiu, Xu Fu

**Affiliations:** 1 Department of Pancreatic and Metabolic Surgery, Nanjing Drum Tower Hospital, the Affiliated Hospital of Medical School, Nanjing University, Nanjing, 210008 China; 2 Department of Pancreatic and Metabolic Surgery, Nanjing Drum Tower Hospital Clinical College of Nanjing University of Chinese Medicine, Nanjing, 210008 China

**Keywords:** Pancreaticoduodenectomy, Rat, Animal model, Postoperative complications, Pancreas, Diseases, Gastroenterology, Medical research

## Abstract

Pancreaticoduodenectomy (PD) is a complex surgical procedure associated with substantial postoperative risk. Despite its clinical significance, the lack of a standardized, cost-effective animal model hinders mechanistic research. Here, we aimed to establish a feasible and safe rat PD model. A simulated PD procedure was performed on Sprague–Dawley rats, including pancreaticojejunostomy (PJ), hepatojejunostomy (HJ), and gastrojejunostomy (GJ). The survival rate, surgical details, enzyme levels (amylase and lipase) and histopathological changes were analyzed. 7-day survival rate was 85.0% (51/60, 95% CI 0.739–0.919). The mean operation duration was 74.9 ± 12.3 min (PJ: 10.0 ± 2.8 min; HJ: 9.7 ± 3.9 min; GJ: 14.8 ± 4.9 min). Histological analysis revealed pancreatic injury at postoperative day (POD) 1, transient acinar-to-ductal metaplasia (ADM) at POD3 and restoration of acinar architecture by POD 7. Immunohistochemistry (IHC) staining demonstrated early acinar apoptosis (C-caspase-3) at POD1-3, CK19 upregulation with reduced amylase at POD3, and re-emergence of acinar markers (amylase) by POD7. Postoperative serum and ascites amylase/lipase levels were statistically elevated and recovered around day 5. This study successfully established a stable and economical rat PD model, providing a practical platform for postoperative mechanistic studies.

**Supplementary Information:**

The online version contains supplementary material available at 10.1038/s41598-025-24072-x.

## Introduction

Pancreaticoduodenectomy (PD) is a high-complexity operation involving multiorgan resection, performed primarily for periampullary or pancreatic head disorders^1^. Despite advancements in surgical technique, morbidity rates remain high (40–64%**)**^2–4^. Numerous clinical studies focused on identifying the risk factors^5^, risk stratification^6^ and novel surgical methods^7^ to mitigate postoperative complications, such as postoperative pancreatic fistula (POPF)^8^ and postoperative acute pancreatitis (PPAP)^9^, their incidence has not substantially decreased over recent decades.

The pathophysiology underlying these complications remains incompletely understood, largely due to the lack of animal models that both physiologically relevant and cost-effective for accurately replicating human PD. Existing canine and porcine models are constrained by cost, ethical considerations, and incomplete anatomical reconstruction, most of which preserve native biliary anatomy or omit gastrojejunostomy^10,11^. Rats are one of the most widely applied animals in diverse experimental pancreatectomy surgical research^12^ with well-defined pancreaticobiliary anatomy. However, a standardized rat PD model incorporating complete organ resection and anastomosis has not been established.

In this study, we developed a rat PD model with complete organ resection and anastomotic reconstruction, closely mimicking human PD. This model provides a practical platform to investigate the pathogenesis of PD-associated postoperative complications, evaluate novel interventions, and train surgeons in complex anastomotic techniques.

## Materials and methods

### Animals

Ten-week-old male Sprague–Dawley (SD) rats purchased from the Qinglongshan Animal Breeding Farm (Nanjing, China) were utilized in this study. SD rats were selected because of their comparable hepatobiliary architecture, sufficient size, simplicity of care and tolerance to surgery. Male animals were applied for minimizing variation in bodyweight and biochemistry. Prior to the experiment, all rats were allowed to acclimatize to the laboratory environment for at least a week and used only when their body weight was 300–350 g. Older animals were not suitable for this operation because of the relatively lower tolerance for surgical trauma. However, young rats weighing less than 250 g were anatomically too small for dissection during this procedure. All rats were housed under specific pathogen-free conditions with a 12 h light–dark cycle, at temperatures ranging from 21 to 25 ℃ and humidity ranging from 37 to 50%, fed standard laboratory chow, and permitted to drink freely. The animal experiments were approved by the Health Research Ethics Board of Drum Tower Hospital of Nanjing University Medical School. All procedures adhered to the Principles of Laboratory Animal Care and the U.S. National Academy of Sciences Guide for the Care and Use of Laboratory. The study was conducted in accordance with THE RULES OF 3R and ARRIVE guidelines^13^.

### Randomization & allocation

Rats were marked with earmark, and randomized table (prepared before surgery by a coordinator independent of surgery and outcome assessment) was used to assign animals to planned assessment time points (POD1, POD3, POD5, POD7) or sham. To minimize selection bias, group assignment for each rat was revealed to the surgeon only after anesthesia induction and baseline monitoring. Cages and sample tubes carried anonymized study IDs without group/time labels. All downstream assessments were blinded: histology sections were ID-coded by the coordinator and reviewed by a pathologists blinded to group/time period after the primary description, and enzyme assays were performed for coordinator-aliquoted, ID-labeled serum and peritoneal-lavage samples by laboratory staff blinded to group/time.

### Anesthesia and pain control

After 12 h preoperative fasting, all animal experiments were placed in an induction chamber containing 1–5% isoflurane via oxygen carrier at 0.8–1.0 L/min until they were recumbent. At the end of induction, the rats were removed from the induction chamber and injected intraperitoneally with pentobarbital for anesthesia maintenance. Depth of anesthesia was determined by respiratory rate, corneal reflex, and response to toe pinching. Analgesia was conducted by Meloxicam (1 mg/kg), which was injected subcutaneously 1 h before surgery.

### Surgical procedure

**Fig. 1:**
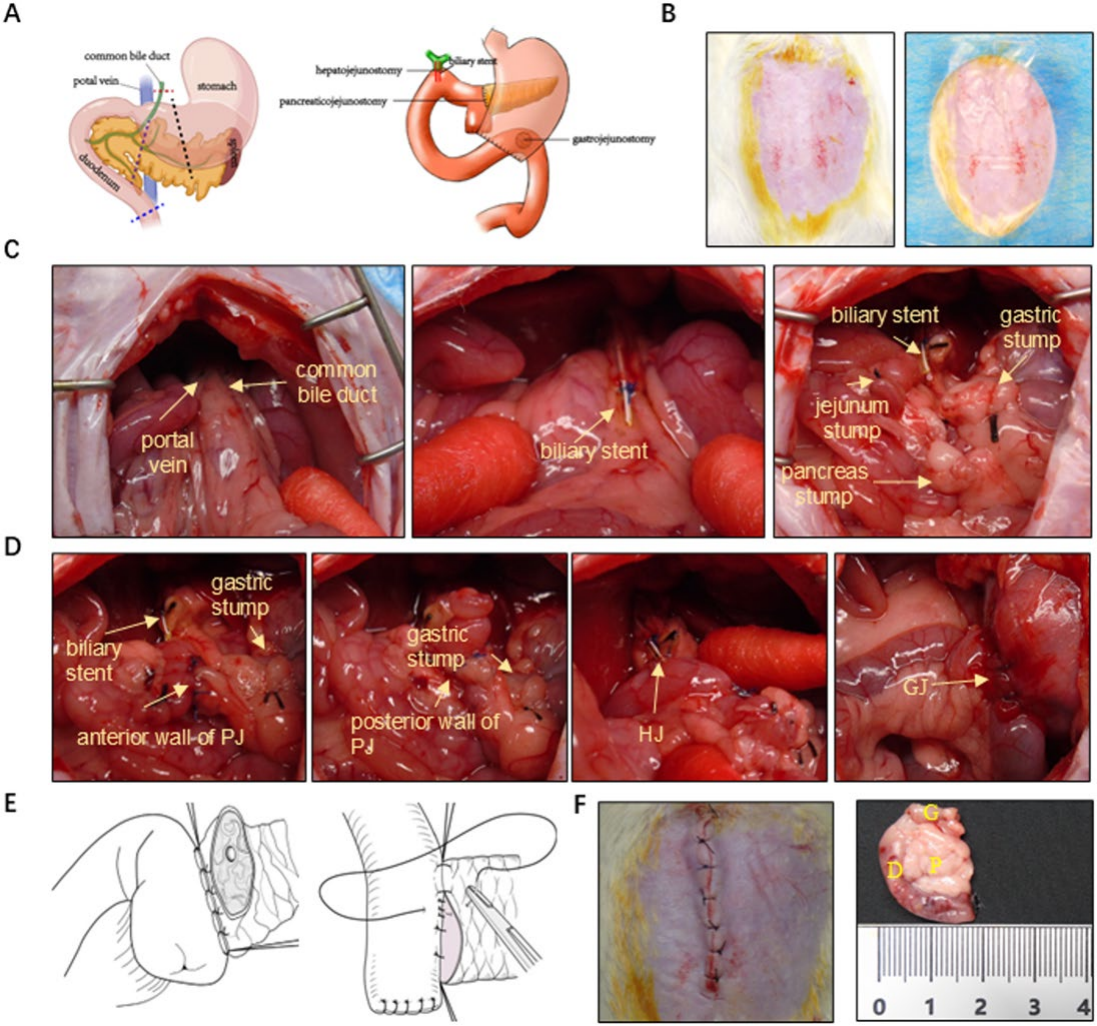
Feasibility of surgical procedure. (**A**) Schematic description of the experimental procedure for PD. The left: planned transection line of common bile duct, stomach, pancreas and duodenum; the right: reconstruction of the digestive tract (PJ → HJ → GJ) after resection; (**B**) Preoperative preparation of rat model. The left: rat in supine position and abdominal skin was shaved and disinfected by iodophor; the right: sterile sheet and surgical adhesive film were performed; (**C**) Technical steps of surgical excision. The left: exposure of CBD and portal vein; the middle: insertion of 24G catheter into the CBD; the right: operation field after specimen removed; (**D**) Procedures of digestive tract reconstruction. The left 1: Anterior wall of PJ; the left 2: posterior wall of PJ; the left 3: establishment of HJ; the left 4: establishment of GJ; (**E**). The left: schematic illustration of the posterior layer of PJ; the right: schematic illustration of the anterior layer of PJ; (**F**) The left: skin was closed by interrupted sutures; the right: specimen was resected en bloc. PD, pancreaticoduodenectomy; CBD, common bile duct; PJ, pancreaticojejunostomy; HJ, hepatojejunostomy; GJ, gastrojejunostomy; D, duodenum; P, pancreas; G, gastric.

All procedures were completed by experienced and skilled pancreatic surgeons who have performed at least 50 PDs annually. The schematic of summarizing the resection, reconstruction order (PJ → HJ → GJ) and biliary stent position was shown in Fig. 1A. After satisfactory anesthesia, animals were immobilized in supine position on the temperature-protected operating table. The abdominal skin was shaved and disinfected by iodophor, sterile sheet was paved (Fig. 1B), a midline skin incision approximately 3 cm was made on the upper abdominal wall at the xiphoid process. All the procedures were performed by sterile technique. The abdomen was incised layer and layer and exposed by a self-retractor before the organs were clearly exposed. The easy way to access the common bile duct (CBD), ventral and dorsal of pancreatic head was to pull the cranial portion of the duodenum caudally. The proximal side of CBD was intubated with approximately 0.5 cm of 24G catheter as a stent and fixed with 6–0 prolene (Polypropylene, Ethicon, USA). while the distal side was suture ligated with 6–0 prolene (Polypropylene, Ethicon, USA) (Fig. 1C). With the stomach pulled anterosuperiorly, left gastroepiploic vessels and left gastric vessels, vessels were ligated and sutured closely with a 5–0 silk thread. The stomach was double ligated and dissected with a 5–0 silk thread approximately 5 mm proximal to the pylorus. Blunt separate the lower margin of pancreas and transverse colon carefully, followed by identifying Treitz ligament along the duodenum. The duodenum was ligated and dissected with 5–0 silk thread on the right side of Treitz ligament. The pancreas was divided into three segments: the biliary, duodenal and gastrosplenic portions^12^. The pancreas on the side to be resected was ligated with 5–0 vichy (Polyglactin 910, Ethicon, USA) thread through the posterior part of the gastrosplenic portion pancreas followed by sharply transection with scissors. To maintain patency of the pancreatic duct, the pancreatic stump was not sutured and bleeding was controlled by compression. Gradual separate, ligate and dissect the biliary and duodenal segments of the pancreas from the caudal side to the cranial side along the right margin of the superior mesenteric vein and portal vein, up to the distal side of the pre-excised CBD, and the specimen was ultimately removed (Fig. 1F).

Reconstruction of the digestive tract was accomplished by the modified Child’s method^14^ (Fig. 1A). About 5–8 cm of the jejunum was lifted to prepare the pancreaticojejunostomy (PJ) and hepatojejunostomy (HJ). Briefly, PJ was conducted by a manual end-to-side, double-layer interrupted anastomosis method (Fig. 1E). The stump of the jejunum was reserved for 0.5 cm to ensure blood supply around the anastomosis site, while the posterior wall of the pancreas was exposed and interrupted sutures of 6–0 prolene (Polypropylene, Ethicon, USA) between the seromuscular layer of the jejunum and the posterior surface of the pancreatic body from the midpoint of the posterior wall to both sides was conducted (Fig. 1D)^15^. Afterwards, the para mesenteric edge of the bowel was opened to a width of approximately half the width of the stump of the pancreas. The anastomosis was completed by placing an anterior row of interrupted sutures of 6–0 prolene (Polypropylene, Ethicon, USA) sutures between the jejunum and the pancreas (Fig. 1D)^16^. Approximately 1 cm from the PJ, a small incision was made by ophthalmic scissors in the wall of the jejunum on the side of the opposite mesenteric rim. The bile stent was inserted into the jejunum at a depth of about 0.5 cm and tightened with a purse-string suture with 6–0 prolene (polypropylene, Ethicon, USA) at a distance of about 0.2 cm from the periphery of the incision (Fig. 1D). An incision of approximately 0.5 cm was made on the posterior wall of the stomach with ophthalmic scissors, and gastrojejunostomy (GJ) was performed by manual side-to-side, monolayer continuous suture anastomosis with 5–0 vichy (Polyglactin 910, Ethicon, USA) thread on the same jejunal loop approximately 6–7 cm from the BJ (Fig. 1D). The abdominal incision was closed in two layers (peritoneum and skin) by continuous sutures with 4–0 vichy (Polyglactin 910, Ethicon, USA) (Fig. 1F).

### Perioperative management

Active warming was provided intra‑ and post‑operatively (surface warming around 38 °C) and all rats were monitored until fully ambulatory. Meloxicam (1 mg/kg) was conducted and re-injected for postoperative analgesia every 24 h postoperatively for 48 h after surgery. All animals received benzyl penicillin sodium at 100,000 IU/kg by intramuscular injection every 24 h for 48 h postoperatively. Immediately after surgery, 2 mL of warmed lactate Ringer solution was administered via the penile vein, and all experimental animals were fasted for 24 h postoperatively. Then 5% glucose-sodium chloride solution was administered orally on postoperative day (POD) 2. On POD 3 and 4, they were fed with soft diet, rat food soaked with water. Solid food was supplied from POD 4. The animals were anesthetized with pentobarbital (60 mg/kg, intraperitoneal injection) and sacrificed by decapitation. Blood samples were collected and pancreas were removed and frozen in liquid nitrogen immediately or embedded for histological assay. Humane endpoints triggering immediate euthanasia by pentobarbital overdose were > 20% weight loss from baseline, persistent hypothermia (< 35 °C), severe lethargy/unresponsiveness, labored breathing, or uncontrolled bleeding.

### Outcomes & definitions

The primary endpoint of the study was 7-day survival after PD. Secondary end points included intraoperative metrics—total operative time, component times for PJ, HJ, and GJ—and postoperative complications. Postoperative complication definitions were adapted from consensus for gastrointestinal anastomotic leak^17,18^ and delayed gastric emptying in pancreatic surgery^19^, complemented by INHAND diagnostic criteria for rodent respiratory pathology and articles on aspiration pneumonia in animal models^20,21^. Gastric leakage was defined by the presence of gastric contents in the peritoneal cavity at necropsy and/or a visible GJ defect with peritoneal soiling. Afferent-loop jejunal necrosis was identified as ischemic necrosis of the reconstructed jejunal loop. Pleural effusion with pulmonary infection was established by purulent tracheobronchial secretions and/or lobar consolidation with pleural effusion at necropsy, clinical tachypnea, and positive culture when available. While, marked gastric dilatation with retained contents together with airway food material/aspiration findings at necropsy was diagnosed as gastric stasis with aspiration.

### Biochemical assay

The rat serum for amylase and lipase detection was taken from the inferior vena cava and centrifuged at 4000 rpm for 10 min at 4 °C, and the upper sera were collected and stored at 80 °C for further analysis. Peritoneal lavage fluid was obtained from the abdominal cavity after irrigation with 4 ml saline, centrifuged to obtain the supernatant and evaluated as ascites. Amylase (100000060, Biosino Bio-technology, China) and lipase (A054-2, Nanjing Jiancheng Bioengineering Institute, China) were analyzed using the respective kits according to the manufacturer’s protocols.

### HE and immunohistochemistry staining (IHC)

Pancreatic tissues were collected and fixed with 10% buffered formalin for 48 h, and paraffin embedded sections were prepared. Slides were stained with hematoxylin and eosin (HE). Two pathologists, blinded to group assignments, scored the severity of edema (grades 0–4), inflammation (grades 0–4), necrosis (grades 0–4) and hemorrhage (grades 0–4) using light microscopy^22^. Histopathologic Schmidt scoring criteria was accordance to assess pancreatic histopathological changes. The percentage of pancreatic acinar ductal metaplasia (ADM) was estimated by the presence of ADM compared to the overall tissue^23^.

For IHC, samples of pancreatic tissues were sectioned at a thickness of 4 mm, processed, and stained with primary antibodies against amylase (3796, Cell Signaling Technology, USA), CK19 (12,434, Cell Signaling Technology, USA) and C-Casp3 (9661, Cell Signaling Technology, USA). The sections were subsequently incubated with an HRP-conjugated goat anti-rabbit IgG (H + L) secondary antibody. Images were captured using an optical microscope (Leica Microsystems, Germany). The intensity of IHC staining was assessed based on a 0 to 3 (0, minimum staining; 1, weak staining; 2, moderate staining; 3, strong staining) scoring method^24^ from six randomly-selected fields of per section using ImageJ software.

### Statistical analysis

Data analysis was conducted by GraphPad Prism 8 (GraphPad Software, San Diego, CA, USA). 7-day survival was summarized with a 95% Wilson confidence interval (CI). Kaplan–Meier curves were generated and compared via the log-rank test. Enzyme measurements and histology assessment were obtained from independent cohorts at sham and POD1/3/5/7 (group sizes: sham n = 8, POD1 n = 6, POD3 n = 7, POD5 n = 7, POD7 n = 6) and reported as mean ± standard deviation (SD) via one-way analysis of variance (ANOVA) with Dunnett’s adjustment versus sham. *P*-values were calculated, and statistical significance was set as *P < 0.05.

## Results

### Feasibility of surgical procedure

The simple schematic of resection, the reconstruction sequence (PJ → HJ → GJ) and biliary stent location were shown in Fig. 1A. After stable and consistent PD models were established, 60 Sprague–Dawley rats with the mean weight 327.9 g were performed for PD model. The mean total operation duration was 74.9 ± 12.3 min. More specifically, the mean time for conducting the PJ, HJ and GJ were estimated to be 10.0 ± 2.8 min, 9.7 ± 3.9 min and 14.8 ± 4.9 min, respectively (Table 1). The survival rate at 7 days after modeling was 85.0% (51/60, 95% CI 0.739—0.919) (Fig. S1B) with no intraoperative death. Nine rats died of postoperative complications. One of them were die on POD 1 because of peritonitis secondary to gastric leakage. Two rats died on POD 2 with the necropsy showed necrosis of afferent loop jejunum. Two rats died of pleural effusion with pulmonary infection on POD 3. Gastric stasis caused 4 rats died on POD 4. The necropsy found large dilated stomachs and signs of aspiration (Table 2). To assess the potential learning-curve effect, we stratified cases chronologically into early (cases 1—30) and late (cases 31—60) stage. Kaplan–Meier curve showed similar 7-day survival between the two groups (*P* = 0.753) (Fig. S1B). Meanwhile, the occurrence of postoperative complication events (*P* = 0.718) and 7-day survival rate were comparable between the two groups (*P* = 0.718) (Fig. S1C).

**Table 1:** Perioperative characteristics of rat-simulated pancreaticoduodenectomy.

Characteristics	All cases (n = 60)
Weight(g)	327.9 ± 14.2
Specimen resection time(min)	28.6 ± 5.9
PJ manipulation time(min)	10.2 ± 2.8
HJ manipulation time(min)	9.7 ± 3.9
GJ manipulation time(min)	14.8 ± 4.9
Total operation time(min)	74.9 ± 12.3

PJ, pancreaticojejunostomy; HJ, hepatojejunostomy; GJ, gastrojejunostomy;

**Table 2:** The complication events after operation.

Complications	Cases	Time of death
Gastric leakage	1	POD 1
Pleural effusion with pulmonary infection	2	POD 3
Necrosis of afferent loop jejunum	2	POD2
Gastric stasis with aspiration	4	POD 4

POD, postoperative days.

### Histological and enzymatic characterization

To evaluate postoperative changes, rats were euthanized on POD 1 (n = 6), 3 (n = 7), 5 (n = 7) or 7 (n = 6). In addition, to rule out the effect of laparotomy on the evaluation of PD rat model development, a sham operation (only laparotomy without any transection of the pancreas) was also performed (n = 8) and sacrificed on POD1 (Table 3). Necropsies were performed in all rats.

**Table 3:** Number of rats sacrificed on each postoperative day.

Complications	Sham	POD1	POD3	POD5	POD7
Rats sacrificed (n)	8	6	7	7	6

POD, postoperative days.

**Fig. 2:**
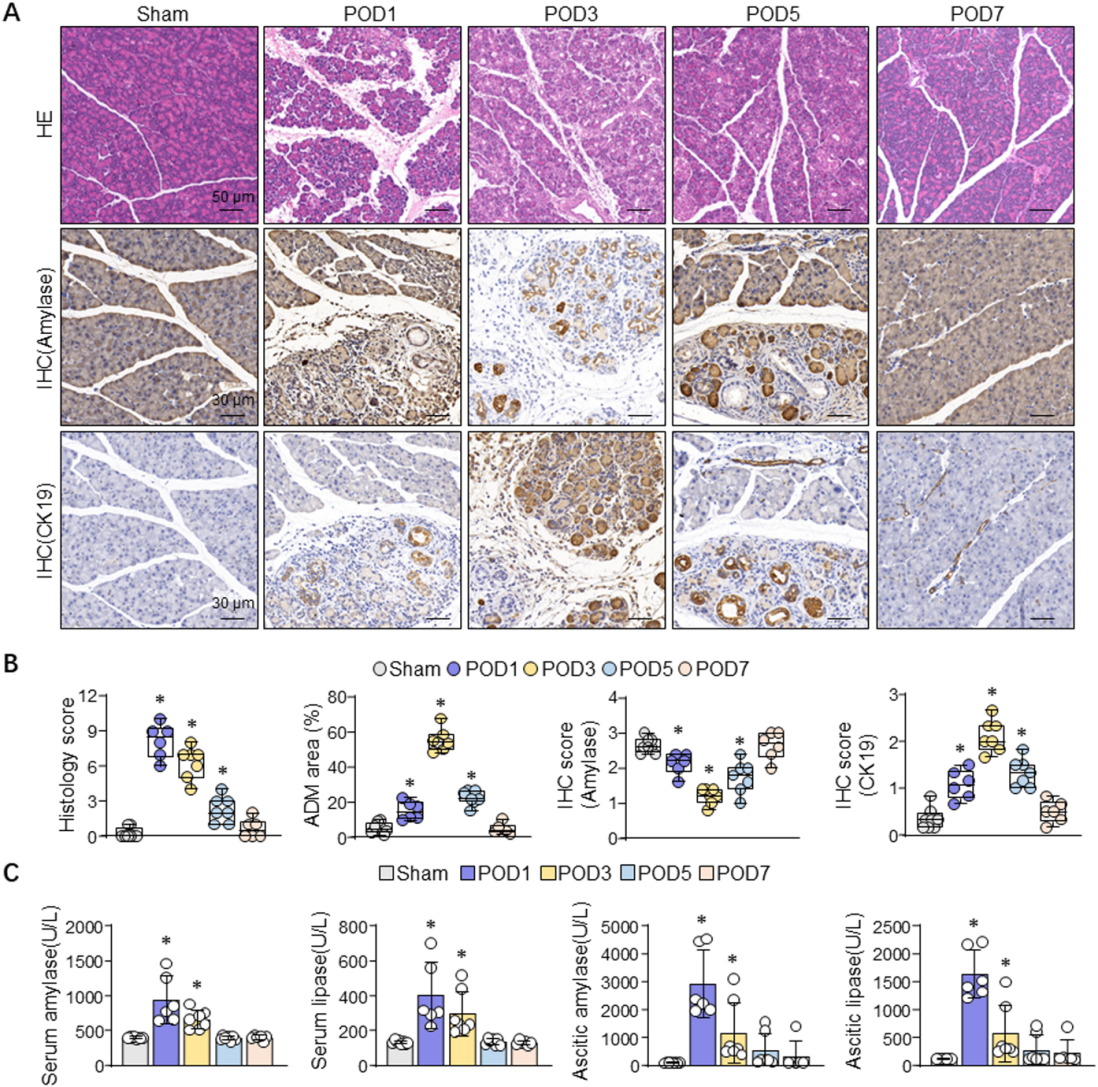
Histological and enzymatic Characterization. (**A**) Representative photomicrographs of pancreatic sections stained by HE and IHC of amylase/CK19 during different POD. (**B**) Pancreatic tissue histopathological score (POD1 *P* = 0.001; POD3 *P* < 0.001; POD5 *P* = 0.004), the area ratio of pancreatic ADM (POD1 *P* = 0.001; POD3 *P* < 0.001; POD5 *P* < 0.001) and the IHC score of amylase (POD1 *P* = 0.023; POD3 *P* < 0.001; POD5 *P* < 0.001)/CK19 (POD1 *P* < 0.001; POD3 *P* < 0.001; POD5 *P* < 0.001) were examined from the experimental rats indicated above. Data were presented as Box-and-whisker plots. *P < 0.05 vs. sham, one-way ANOVA (Group size: sham n = 8, POD1 n = 6, POD3 n = 7, POD5 n = 7, POD7 n = 6). (**C**) Serum and ascitic amylase (Serum: POD1 *P* < 0.001; POD3 *P* = 0.001; Ascitic: POD1 *P* < 0.001; POD3 *P* = 0.049)/lipase (Serum: POD1 *P* < 0.001; POD3 *P* = 0.010; Ascitic: POD1 *P* < 0.001; POD3 *P* = 0.042) levels were examined from the experimental rats indicated above. Data were presented as mean ± SD. *P < 0.05 vs. sham, one-way ANOVA (Group size: sham n = 8, POD1 n = 6, POD3 n = 7, POD5 n = 7, POD7 n = 6). HE: haematoxylin & eosin; IHC: immunohistochemistry; POD: postoperative day; ADM: acinar ductal metaplasia.

To investigate the pathological process following PD, we collected the pancreas at the different time points within one week. HE evaluation of the pancreas remnant revealed classic features pancreatic tissue injury—marked signs of interstitial edema, acinar necrosis and leukocyte infiltration at POD1. And normal acini were lost and replaced by progressive ADM with infiltration of inflammatory cells at POD3. Meanwhile, typical ADM structure gradually recovered to normal acini around POD7 (Fig. 2A and B). Immunohistochemistry staining for amylase (acinar), CK19 (ductal/ductular) and cleaved caspase-3 (apoptosis) demonstrated reduced amylase at POD1-3 with recovery by POD7, transient CK19 upregulation in ADM at POD3 returning toward baseline by POD7, and C-Casp3–positive acinar cells peaking at POD1-3 and sparse by POD7 (Fig. 2A, B and S1A).

Mean serum amylase and lipase levels were, respectively, 400.7 ± 14.6 and 133.5 ± 9.7 U/L on sham, 940.6 ± 336.4 and 400.9 ± 186.5 U/L on POD1, 661.1 ± 132.7 and 297.7 ± 125.7 U/L on POD3, 394.2 ± 32.3 and 137.6 ± 17.6 U/L on POD5, 399.4 ± 32.4 and 130.2 ± 12.3 U/L on POD7. Mean ascitic amylase and lipase levels were, respectively, 103.5 ± 16.9 and 121.6 ± 5.7 U/L on sham POD1, 2915.3 ± 1218.0 and 1635.9 ± 439.2 U/L on POD1, 1169.2 ± 1087.9 and 574.9 ± 496.5 U/L on POD3, 526.8 ± 607.7 and 280.1 ± 260.3 U/L on POD5, 335.0 ± 524.9 and 225.3 ± 229.9 U/L on POD7 (Fig. 2C). Both serum or ascites amylase and lipase increased in the early postoperative days (POD1 and 3) compared to sham group, while returned towards a relative normal level around POD5.

## Discussion

In this study, we successfully established a feasible, safe and cost-effective rat model of PD for the first time. This model provided a valuable experimental platform investigating perioperative physiological responses (analgesia, endocrine/exocrine function) and pathophysiology of PD-associated complications (e.g., POPF, PPAP)^25–27^.

PD, which is considered the most effective surgical treatment for treating disorders located near the head of the pancreas and the Vater’s ampulla, involves the resection of multiple organs and the reconstruction of several digestive tract, making it a high-risk endeavor with high incidence of postoperative complications^2–4^. During the past decades, some researchers constructed porcine^11^ and canine^10,28,29^ models of PD to investigate the gland function and new methods of PJ or HJ anastomosis, however the models reported previously lack uniform standardize, meanwhile, the application of these large animals involve challenges in terms of cost, administrative barriers and ethical considerations. For instance, Peng et al.^11^ conducted a porcine model investigating the pancreatic exocrine function by performing PJ anastomosis between dilated pancreatic duct and jejunum. However, the model only ligated the pancreatic duct which result in dilatation of the pancreatic duct for PJ without performing digestive tract reconstruction of the HJ and GJ. Khajanchee et al.^30^ caried out an animal experiment of PD by porcine. They held the duodenum proximal and distal to the pancreas, retained the CBD and resected a portion of the pancreas for an end-to-end PJ. In such a PD model, the bile duct anatomy was not disrupted and the HJ was not performed according to the steps in the conventional PD procedure. A canine training model for digestive tract reconstruction of PD was established by Yang et al.^10^. However, this model did not adequately simulate the pathophysiologic alterations after PD in human since the head of the pancreas and duodenum were not completely removed and no GJ anastomosis was performed. Briefly, prior large-animal models (porcine/canine) have often focused on a single anastomosis (e.g., PJ or GJ) or partial reconstructions and used brief postoperative observation periods. In contrast, we established a rat model which performs PD with complete reconstruction (PJ + HJ + GJ), enabling concurrent assessment of pancreatic, biliary, and gastric anastomoses in a scalable small-animal setting. Because of the differences of follow-up duration, perioperative care and outcome definitions among published studies, the occurrence of postoperative complications and mortality rates were varied wildly. Accordingly, our study provides a feasible and stable platform for further investigation on pathophysiological changes after PD.

There are few reports of rat models about PD in the literature. Utria et al.^31^ established an animal model to simulate pancreatic fistula after PD in rats by ligating the CBD and sharply transected the common pancreatic duct. The anatomical structure of the rat pancreas which consisted by the biliary, duodenal and gastrosplenic portions was previously studied in detail by Kara et al.^12^ The size and anatomic variation of pancreatic anatomy offered several technical challenges but is a promising model for the study of PD. In the PD model we established, the remnant pancreatic duct was excessively small, we performed an effective and safe PJ anastomosis by performing an end-to-side anastomosis of the pancreatic stump to the side wall of the jejunum. The strength of this anastomosis is that it is less likely to tear the pancreas, while the anterior and posterior walls of the anastomosis are precisely sutured under direct observation. The mean diameter of CBD in rat was 1.01 mm according to reported previously^12^, which causes great difficulty in the HJ anastomosis. 24G catheter was applied as a biliary stent during HJ and purse-string suture was performed for fixing the stent. The reconstruction of biliary tract by stent was widely accepted in the rat liver transplantation^32,33^. The superiority of such anastomosis lied in the fact that the biliary reconstruction is precise and the suture was hard to entangle with knots left outside the anastomosis. At the same time, the model is completed by pancreatic surgeons with basic surgical skills in suturing and precise dissection, which guarantees less time-consuming and less traumatizing for rats to be manipulated.

In current study, we observed that serum amylase/lipase peaked at POD1 and approached sham by POD5-7, whereas ascitic levels declined more slowly and were numerically higher than sham at POD7 without a statistically significant difference. In parallel, immunohistochemistry staining showed a transient ADM peak at POD3 (CK19 upregulation with reduced amylase) and re-emergence of acinar identity by POD7, with cleaved caspase-3 indicating early acinar apoptosis at POD1. This combination supports a self-limited acinar injury with systemic biochemical recovery by POD5-7 and a local peritoneal compartment in which enzymes clear more slowly and/or are replenished by low-grade exudation from healing anastomoses. The overall trajectory—transient pancreatic injury and ADM with resolution and redifferentiation—was consistent with mechanisms described in pancreatitis models^23^, while the persistent ascitic enzyme signal may highlight the distinct kinetics of the peritoneal space after PD. Meanwhile, the mortality rate of current model remains as high as 15%, likely due to the fact that PD involves the resection of multiple organs and gastrointestinal reconstruction, while rats have a lower tolerance for surgical trauma compared to larger animals such as porcine and canine. Overall, the model enables an in-depth study of perioperative pathophysiological alteration (e.g. endocrine/exocrine pancreatic functions, postoperative complications) during PD. Furthermore, given that PD is a technically demanding procedure associated with significant risk, the surgeon’s technical mastery is paramount to ensuring operative safety. Consequently, establishing a validated, reproducible, and physiologically relevant animal training model is critical for enhancing the safety of gastrointestinal reconstruction during PD. The development and utilization of current rat model can, to some extent, enhance the proficiency of young surgeons in performing requisite anastomotic techniques.

The current study has several limitations. First, the technical complexity of the model necessitates specialized training and is better performed or guided by an experienced surgeon. Second, the potential or specific mechanism by which ADM is converted needs to be further investigated. Finally, the long-term effects in rats after PD are unknown which may influence the subsequent application of this model for mechanistic investigations.

## Conclusions

This rat PD model is reproducible and clinically relevant, offering a robust platform for investigating postoperative pathophysiology and refining surgical techniques.

## Supplementary Information

Below is the link to the electronic supplementary material.

Supplementary Material 1.

## Data Availability

The datasets generated and/or analyzed during the present study are not publicly available due to patient privacy concerns, but are available from the corresponding author upon reasonable request.
