# Supplementary material for: Development of a novel rat model for pancreaticoduodenectomy
Source: Sci Rep. 2025 Nov 18;15:40458. doi: 10.1038/s41598-025-24072-x (PMC12627809; doi:10.1038/s41598-025-24072-x)
Supplement: Supplementary file 1 — Supplementary Material 1. [file 41598_2025_24072_MOESM1_ESM.docx]

**Supplemental Figure S1**


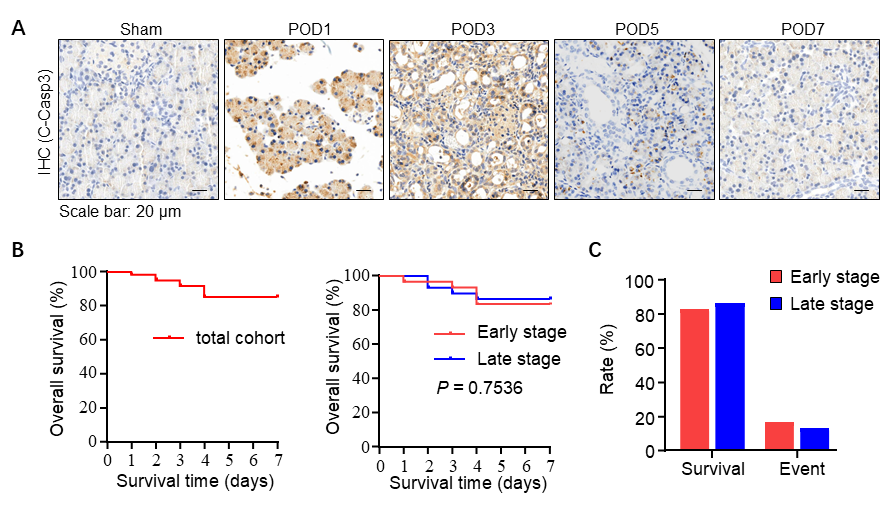


**Supplemental Figure S1.** (A) Representative photomicrographs of pancreatic sections stained by immunohistochemistry (IHC) for amylase/CK19 during different postoperative day (POD). (B) Kaplan-Meier survival analysis for the total cohort (the left) and for the early/late stage cohort (the right) (n=30 per cohort, *P* = 0.7546 by log rank test). (C) 7-day survival rate (*P* = 0.718) and occurrence of postoperative complication events (*P* = 0.718).
